# Non-Steroidal Anti-Inflammatory Drugs: Pharmacokinetics and Mitigation of Procedural-Pain in Cattle

**DOI:** 10.3390/ani11020282

**Published:** 2021-01-22

**Authors:** Brooklyn K. Wagner, Emma Nixon, Ivelisse Robles, Ronald E. Baynes, Johann F. Coetzee, Monique D. Pairis-Garcia

**Affiliations:** 1 Department of Population Health and Pathobiology, College of Veterinary Medicine, North Carolina State University, Raleigh, NC 27606, USA; bwagner2@ncsu.edu (B.K.W.); enixon@ncsu.edu (E.N.); irobles@ncsu.edu (I.R.); rebaynes@ncsu.edu (R.E.B.); 2 Department of Anatomy and Physiology, College of Veterinary Medicine, Kansas State University, Manhattan, KS 66506, USA; jcoetzee@vet.k-state.edu

**Keywords:** cattle, NSAID, flunixin, meloxicam, aspirin, pain control

## Abstract

**Simple Summary:**

Castration and disbudding, common husbandry procedures used in cattle livestock production industries, are recognized as being painful. In the United States (U.S.), these procedures are frequently performed without pain relief. Although non-steroidal anti-inflammatory drugs (NSAIDs) are used in food-animal production systems, no such pain relief drugs are federally approved in the U.S. for controlling procedural pain in cattle to date. Recent increases in consumer, retailer and producer commitment to improving the welfare of food-animals necessitate a closer look into pain control efforts for livestock. Therefore, this review comprehensively evaluated existing literature to summarize three NSAIDs (meloxicam, flunixin and aspirin) (1) pharmacokinetics and (2) administration outcome in regard to pain control during castration and disbudding procedures, in cattle. The sample size contained notable variability and a general deficiency of validated and replicated methodologies for assessing pain in cattle represent on-going challenges. Future research should prioritize replication of pain assessment techniques across different experimental conditions to close knowledge gaps identified by the present study and facilitate examination of the effectiveness of pain relief drugs.

**Abstract:**

Common routine management practices in cattle, such as castration and disbudding, are recognized as being painful. In the United States (U.S.), these procedures are frequently performed without pain mitigation and there are currently no drugs federally approved for such use. Non-steroidal anti-inflammatory drugs, such as meloxicam, flunixin meglumine and aspirin, are the most commonly used analgesics in U.S. food-animal production systems. However, the body of research investigating the effectiveness of these pharmaceuticals to control pain in cattle at castration and disbudding has not been comprehensively evaluated. Therefore, this review examined existing literature to summarize meloxicam, flunixin and aspirin (1) pharmacokinetics (PK) and (2) administration outcome in regard to pain control during castration and disbudding procedures, in cattle. Following systematic searches and screening, 47 PK and 44 publications were extracted for data and are presented. The sample size contained notable variability and a general deficiency of validated and replicated methodologies for assessing pain in cattle remain substantial challenges within this research area. Future research should prioritize replication of pain assessment methodologies across different experimental conditions to close knowledge gaps identified by the present study and facilitate examination of analgesic efficacy.

## 1. Introduction

Common husbandry practices in cattle, such as castration, disbudding and branding, are recognized as being painful for the animal [1] and present a growing animal welfare concern for livestock production industries. In the United States (U.S.), such procedures are frequently performed without pain mitigation [2,3]. Although terminating the use of such painful procedures would improve animal welfare by eliminating procedure-associated pain, logistical factors (e.g., human safety, facility design, cost, etc.) in U.S. production systems currently necessitate the use of these procedures. Therefore, minimizing pain through pharmaceutical interventions is the next practical step.

Although there is evidence to suggest that some available drugs relieve pain in cattle, lack of regulatory approval by the U.S. Food and Drug Administration (FDA; regulatory agency in the U.S. responsible for assessing drug safety and efficacy) may be an impediment to practical on-farm application for cattle producers [4]. To date, there are no pharmaceutical pain relief options approved by the U.S. FDA specifically for use in cattle undergoing common husbandry procedures such as castration and disbudding. Furthermore, obtaining such approval remains challenging as drug efficacy must be proven (i.e., must scientifically demonstrate a reduction in pain) via validated methodologies [5].

Non-steroidal anti-inflammatory drugs (NSAIDs; e.g., meloxicam, flunixin meglumine, aspirin) are the most commonly used analgesics in U.S. food-animal production systems [6,7]. Flunixin meglumine is approved for general use as an anti-pyretic and anti-inflammatory [8,9] and transdermal flunixin is approved for controlling fever associated with bovine respiratory disease and pain specifically associated with foot rot in cattle [6]. Therefore, any use of this NSAID outside these indications would be considered extra-label drug use (ELDU) and must be guided by a veterinarian in accordance with the Animal Medicinal Drug Use Clarification Act [9,10]. Meloxicam, though approved for use in cattle in the European Union and Canada, is not approved for use in cattle in the U.S. Similarly, and despite its wide use for controlling fever and minor pain, aspirin maintains no formal U.S. FDA approval and is not recommended for use in food animals by the Food Animal Residue Avoidance Databank (FARAD) [8,9]. Lack of U.S. FDA approval dictates that although extra-label use is not prohibited, any use of these drugs to mitigate procedural pain would be considered ELDU, would require veterinarian oversight and an extended withholding period to avoid residues in meat and milk.

Understanding the impact that pharmacokinetic properties have on pain mitigation efficacy in cattle is an important factor for moving forward with effective pain management protocols on-farm. By having access and better understanding of pharmacokinetic data, veterinarians are able to more effectively develop ideal dosing regimens for optimal pain relief and identify optimal drug category selection and administration routes that are less impacted by external factors such as sex, age and health status. Even on a global level, where analgesic used is approved for veterinarian use, access to pharmacokinetics (PK) data will allow these veterinarians to make better decisions for mitigating pain across diverse production systems.

In order to address this growing need, science-based guidelines identifying effective pharmaceutical protocols to mitigate procedural pain in cattle are needed. To date, the body of research investigating pharmacokinetic properties of meloxicam, flunixin and aspirin as pain control drugs for use in cattle undergoing routine husbandry procedures has not been comprehensively evaluated. Therefore, this review aimed to examine the existing literature of meloxicam, flunixin and aspirin to provide a useful summary of (1) pharmacokinetics, and (2) administration outcome in regard to pain control during castration and disbudding procedures, in cattle.

## 2. Materials and Methods

### 2.1. Pharmacokinetics

Pharmacokinetic (PK) data used in this study encompassed a 35-year period (1984–January 2020) and were collected using systematic search criteria in conjunction with both the Food Animal Residue Avoidance Databank (FARAD) [11,12] and PubMed. Briefly, FARAD represents the most extensive and accessible compilation of PK information for veterinary drugs available to date [11]. Within the FARAD system, an advanced search method was used such that the drug of interest was entered, and all relevant name variations were selected (e.g., flunixin and flunixin meglumine), followed by species (e.g., bovine, cattle, etc.) and desired matrices (e.g., plasma). A second search was performed using PubMed to ensure that all relevant PK studies were identified and included. Search criteria and keywords included: “pharmacokinetics”, “plasma concentration(s), “plasma level(s)”, half(-)life, “peak concentration(s)”, “absorption”, “bioavailability”, “AUC”, “C_max_”, “T_max_”, “volume of distribution”, “NSAID”, “meloxicam”, “flunixin”, “aspirin”, “salicylic acid”, “analgesi(a)(c)”, “cattle”, “cow(s)”, “heifer(s)”, and “cal(f)(ves)”. Publications were included or excluded based on inclusion and exclusion criteria including: (1) population (only cattle included) and drug (only meloxicam, flunixin or aspirin included); (2) matrix considered (only plasma included); (3) major PK parameter reported (at least one of the following must be reported to be included: half-life, clearance or volume of distribution); (4) review papers or duplicate data (excluded).

### 2.2. Drug Administration Outcome: Systematic Literature Review

#### 2.2.1. Search Strategy and Screening Process

A systematic literature search was conducted in collaboration with the William Rand Kenan Jr. Library of Veterinary Medicine at North Carolina State University. Published scientific literature between 1990 and 31 December 2019 was searched in five electronic databases: Agricola (EBSCO), CAB Abstracts, PubMed, ProQuest Dissertations and Theses Full Text, and Web of Science (all databases). The search performed aimed to collect information on NSAID administration outcomes in both cattle and swine, therefore initial search result publication numbers represent an overestimation when only considering cattle as with the present study. Both controlled vocabulary and keywords were used for three main concepts including (1) pain control (e.g., “pain management”, “alleviat(e)(ion)”, “mitigate(e)(ory)(ion)”); (2) cattle (e.g., “cow(s)”, “cal(f)(ves)”, “bovine”); and (3) drugs (e.g., “NSAIDs”, “meloxicam”, “flunixin”).

Search results were systematically screened by two independent researchers using Covidence online platform (Covidence Systematic Review Software, Veritas Health Innovation, Melbourne, Australia). Researcher 1 (MDP-G) is an Associate Professor who holds a DVM and a PhD, with expertise in pain in livestock and animal welfare on a global scale. Researcher 2 (BKW) is a postdoctoral research associate in animal welfare with a PhD and expertise in cattle health, physiology and welfare.

Following manual removal of duplicate studies, ‘Titles and Abstracts’ were screened for relevance using inclusion and exclusion criteria (criteria details can be found in Appendix A). Next, publication ‘Full Texts’ were screened and included or excluded based on the following criteria: (1) population (only cattle included), drug (only meloxicam, flunixin or aspirin included), and procedure (only castration and disbudding included); (2) number of concurrent procedures (excluded publications using > 1 procedure); (3) review papers, abstract/conference proceedings, or texts that were unavailable in English (excluded); (4) presence of a control treatment (mandatory for inclusion).

#### 2.2.2. Data Extraction

**Figure 1:**
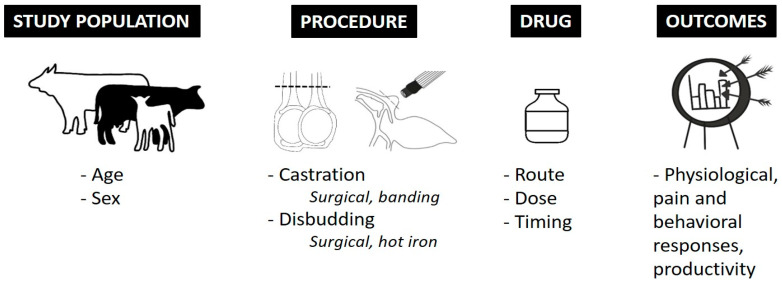
Information systematically and manually collected from each publication.

Following the screening process, one trained researcher (BKW; a postdoctoral research associate with expertise in both cattle production and animal welfare) extracted data from all remaining articles. Specific study information, outlined in detail in Figure 1, was collected and organized systematically by the same trained researcher. Positive (effective) or negative (not-effective) administration outcome was determined by BKW based on efficacy statements made by authors of these publications as demonstrated by a *P*-value less than 0.05 between drug of interest and control treatment. Further qualitative analysis of publications was not performed by BKW or MDP-G.

Using this data, measured outcomes were categorized (Table 1) and descriptively quantified to enrich data visualization and facilitate result interpretation.

## 3. Results and Discussion

### 3.1. Pharmacokinetics

A total of 95 reports were identified and screened using criteria described in the Materials and Methods resulting in data being collected from 49 publications (FARAD, *n* = 43; PubMed, *n* = 6). In total, 99 data sets were considered in the present study across 15 publications investigating meloxicam (datasets, *n* = 25), 27 publications investigating flunixin (*n* = 62) and five publications investigating aspirin (*n* = 8). Pharmacokinetic profiles for meloxicam and flunixin are presented by administration route (e.g., intravenous, subcutaneous, etc.) in Table 2.

For aspirin, intravenous and oral administration routes were used in five and three datasets, respectively. In addition, varying aspirin compounds (acetylsalicylic acid, *n* = 5; sodium salicylate, *n* = 3) and dosages (ranging from 26–100 mg/kg) were used. Not all studies reported half-life (T_1/2_), clearance (CL or CL/F) or volume of distribution (V_d_ or V_d_/F); therefore, and given the small sample size, aspirin data are not included in Table 2.

Pharmacokinetic data compiled and presented here represents a comprehensive overview of all published PK literature available between 1975 and August of 2020 for meloxicam, flunixin and aspirin. Notably, existing research exhibits a tendency for a route of administration and such tendencies differ by drug. Specifically, the majority of publications in meloxicam utilized oral (PO; i.e., *per os*) drug administration, while intravenous (IV) administration was favored for flunixin. The lower cost and wider availability associated with PO meloxicam, compared with injectable meloxicam may contribute to the PO administration route being heavily favored. Furthermore, IV flunixin is cost effective and avoids risks associated with intramuscular (IM) injections (e.g., Clostridial myositis [13]). In addition to cost, work conducted by Robles and colleagues (2020) found that producers identified logistics surrounding administering a drug as a major challenge given some of these drugs would require multiple handling events for the animals and increased risk for carcass condemnation if a needle were to break into the muscle.

Additionally, the present study revealed a large degree of variability (i.e., large min-max range) in drug half-life, time to maximum concentration (T_max_) and volume of distribution for most administration routes across both meloxicam and flunixin, with the exception of IV meloxicam and SC flunixin. Given that many factors play a role in drug metabolism, including but not limited to age [14,15], sex [16], genetics [15,16], disease or health status [15], and physiological state (e.g., pregnant of lactating) [15], such differences between studies likely contribute to this variability. The intent of reporting pharmacokinetic parameters was to provide evidence for a possible source of this variability and provide a clear putative of both the pharmacokinetics and drug administration outcomes. Unfortunately, many of the publications lacked information on important covariates (age, weight, sex, genetics, health status, physiological status) that may directly drive such variation in effectiveness. Given this, these limitations precluded the ability to conduct a thorough PK/pharmacodynamics analysis, thus highlighting some of the challenges with both the quality and quantity of data available for this review.

Moving forward, additional PK research, especially for aspirin and salicylate, is needed to determine which of the above covariates influence this variability in PK parameters and dose-response relationships for these NSAIDSs. This will ultimately determine whether dosage regimen adjustments are needed to improve efficacy of these drugs.

### 3.2. Pain Mitigation in Cattle

**Figure 2:**
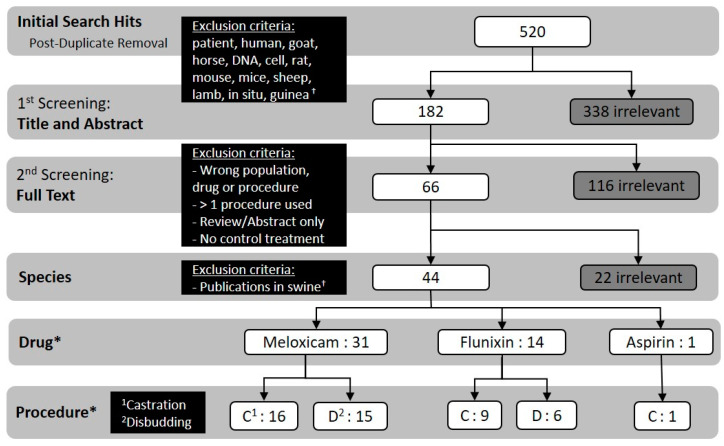
Flowchart detailing the screening process and numerical outcome of each step (i.e., how many publications qualified for a given step based on the previous step’s criteria). ^†^ The present study was part of a larger study that also aimed to collect similar information in swine, therefore swine publications (*n* = 22) qualifying for full text screening were removed at the “species” screening step. * Numbers in these rows do not align mathematically with previous rows due to the presence of publications that reported data for either both flunixin and meloxicam or both castration and disbudding procedures (independent treatments, e.g., procedures not performed concurrently).

A total of 520 documents from the literature were identified and screened using criteria described in the Materials and Methods. Of these, 43 publications were included based on population, drug, procedure, publication type and treatment criteria (Figure 2).

#### 3.2.1. Meloxicam and Flunixin: Castration.

**Figure 3:**
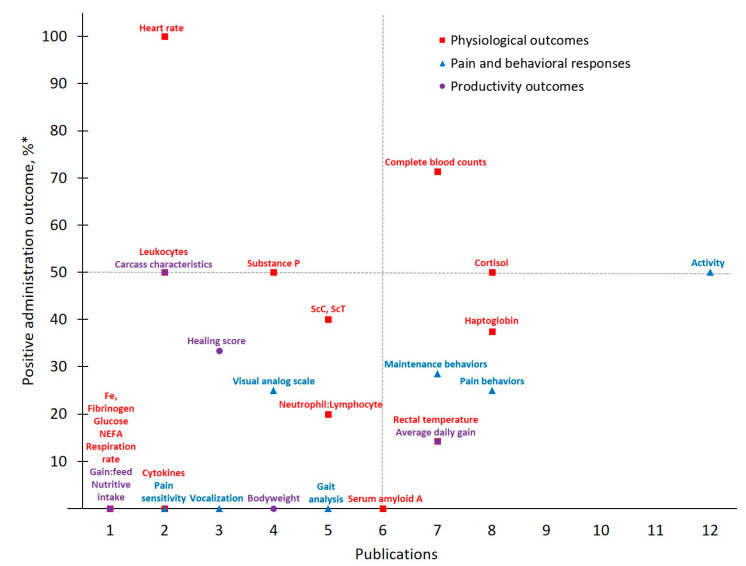
The number of publications that measured a given outcome and the percentage of publications that demonstrated meloxicam to have positive administration outcomes for cattle at castration. Positive administration outcome was determined based on efficacy statements made by authors of these publications as demonstrated by a *P*-value less than 0.05 between meloxicam and control treatment. Outcomes measured: NEFA = non-esterified fatty acids; ScC, ScT = scrotal circumference and temperature. *$\;\mathrm{Positive}\;\mathrm{administration}\;\mathrm{outcome}\;\%=\frac{\#\;\mathrm{of}\;\mathrm{publicationsthat}\;\mathrm{demonstrated}\;\mathrm{positive}\;\mathrm{administration}\;\mathrm{outcome}\;\mathrm{via}\;x\;\mathrm{outcome}}{\#\;\mathrm{of}\;\mathrm{publications}\;\mathrm{that}\;\mathrm{measured}\;x\;\mathrm{outcome}}\times 10$.

**Figure 4:**
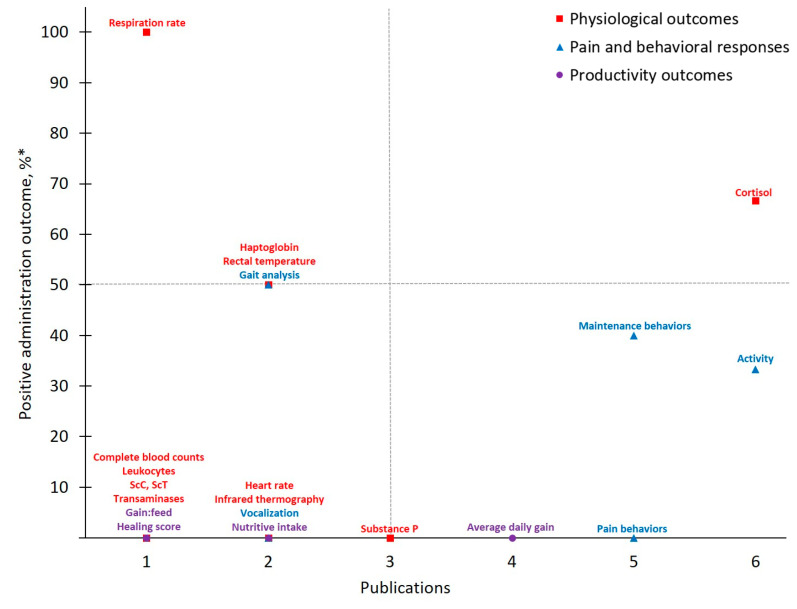
The number of publications that measured a given outcome and the percentage of publications that demonstrated flunixin to have positive administration outcomes for cattle at castration. Positive administration outcome was determined based on efficacy statements made by authors of these publications as demonstrated by a *P*-value less than 0.05 between flunixin and control treatment. Outcomes measured: ScC, ScT = scrotal circumference and temperature. ^*^ $\mathrm{Positive}\;\mathrm{administration}\;\mathrm{outcome}\;\%=\frac{\#\;\mathrm{of}\;\mathrm{publicationsthat}\;\mathrm{demonstrated}\;\mathrm{positive}\;\mathrm{administration}\;\mathrm{outcome}\;\mathrm{via}\;x\;\mathrm{outcome}}{\#\;\mathrm{of}\;\mathrm{publications}\;\mathrm{that}\;\mathrm{measured}\;x\;\mathrm{outcome}}\times 10.$

Data collected from publications that explored castration pain in cattle administered meloxicam (MEL-CAST; *n* = 16) or flunixin (FLU-CAST; *n* = 9) are presented in Table 3 and Table 4, respectively. Eleven publications reported that meloxicam demonstrated 16 out of 29 positive administration outcomes for controlling castration pain. Seven publications reported that flunixin demonstrated seven out of 21 positive administration outcomes for controlling castration pain. Publication totals (i.e., # of publications that measured a given outcome) and positive administrate outcome rates for each measured outcome in castrated cattle administered meloxicam or flunixin are presented in Figure 3 and Figure 4, respectively. Additionally, of the publications that reported calf age, 60% of studies investigating castration reported using animals ≥ six months of age, while 27 and 13% reported using calves ≤ two months of age and between two and six months of age, respectively.

Meloxicam and castration represent the most frequently investigated drug-procedure combination considered by the present study, yielding 29 total outcomes measured across 16 publications. *Activity*, categorized under ***Pain and Behavioral Responses***, was the most frequently assessed outcome (publications, *n* = 12). However, only six publications reported that meloxicam mitigated castration pain (*p* < 0.05) based on *Activity* assessments, resulting in a 50% positive administration outcome. Variability in both which specific activity outcomes are measured (e.g., chute behaviors, leg movements, time spent lying or standing, etc.) and how they are assessed (e.g., pedometers, observation and scoring, etc.) may contribute to this non-descript administration outcome. For example, while some chose to assess *Activity* using accelerometers [27], others relied on behavioral observations of pen (e.g., walking) [31] or chute [28] activities. Such inconsistencies further limit the development of robust conclusions and recommendations for pain management in cattle.

Contrasting *Activity* findings, some ***Physiological Outcomes*** demonstrated more positive administration outcomes with low measurement frequency. Of note for MEL-CAST, *Heart rate* stands out with the greatest positive administration outcome (100%; i.e., measures determined to demonstrate positive administration outcome if significant (*p* ≤ 0.05) differences between analgesia and control treatments were identified). Heart rate and heart rate variability remain attractive options due to their objectivity and ability to be measured non-invasively. Increases in these parameters have been reported in cattle undergoing various procedures including castration [19,27], disbudding [46,47] and branding [48]. However, low measurement frequency inhibits reliable result interpretation regarding both meloxicam’s ability to mitigate castration pain and *Heart rate* as a consistent indicator of pain in cattle. Access and cost limitations of heart rate monitoring equipment, though having become more affordable and thereby more accessible over time [49], may be restricting the existing body of work utilizing this physiological outcome. Furthermore, logistical challenges associated with on-farm use may play a role. For example, of the two MEL-CAST studies that measured *Heart rate*, both were conducted on research farms rather than on commercial operations [19,27], potentially indicating challenges associated with monitoring heart rate under less controlled conditions [50]. Moving forward, more practical options for monitoring *Heart rate* in commercial settings (e.g., reduced equipment costs, safe and easy application) may help facilitate methodology validation for *Heart rate* as an indicator of pain in cattle.

In addition to *Heart rate*, experts often rely on various physiological parameters as direct indicators of biological functioning, one of the three schools of animal welfare [51], and this tendency was evident across MEL-CAST publications. However, greater assessment frequency did not result in improved positive administration outcomes, given that 14 out of 16 total ***Physiological Outcomes*** measured demonstrated positive administration outcome rates ≤ 50%. Nevertheless, investigator inclination to rely on physiological measures persisted across all drug and procedure combinations identified by the present study. Contributing factors may include researcher confidence with the objective nature of physiological assessments, or the relative ease of measuring most physiological parameters once blood is collected.

Publications investigating flunixin and castration pain in cattle totaled just half of the number of publications identified for meloxicam. Of the 21 different outcomes measured across these nine reports, ***Physiological Outcomes*** comprised the majority (*n* = 12). Of these categorized parameters, 83% were measured on ≤ two instances and the greatest positive administration outcome identified was *Respiration rate* (100%). In contrast, of the five ***Pain and Behavioral Responses*** measured, 60% were assessed by ≥five publications. However, these more frequently measured outcomes resulted in a low positive administration outcome (25%). As with earlier discussion for meloxicam, behavioral outcomes assessed in FLU-CAST publications were variable in regard to what, and how, specific parameters were measured. These inconsistencies may contribute to the low positive administration outcome rates observed for *Activity*, *Maintenance behaviors*, *Pain behaviors* and *Vocalizations*. For example, Currah et al. [33] utilized subjective visual observations to evaluate animal behavior in the chute and demonstrated no effect of flunixin on chute activity [33]. In contrast, Repenning et al. [28] evaluated chute activity objectively with the use of timers and cameras and ultimately reported flunixin to mitigate pain in castrated calves based on duration in the chute and exit velocity [28]. Such discrepancies in assessment method, paired with the limited availability of research for FLU-CAST to date, make it difficult to determine if drugs, in this case flunixin, are in fact effective.

In comparison to ***Pain and Behavioral Responses***, *Cortisol* offers more consistency and is well studied to date as a standard measure of the body’s response to various challenges [52]. In the present study, investigators favored this ***Physiological Outcomes*** as evidenced by *Cortisol* being measured with the greatest frequency. *Cortisol* acts as the final effector of the hypothalamic-pituitary-adrenal axis (i.e., mammalian stress response) and its measurement can provide physiological insight into an animal’s homeostatic state [52,53]. Procedural events such as castration result in increased cortisol concentrations immediately post-incision in response to acute pain [54,55] and peak cortisol concentrations approximately 30-min post-procedure [56]. Due to its assessment ease and pervasiveness in existing literature, as well as positive administration outcome rates for meloxicam (50%, *n* = 8) and flunixin (67%, *n* = 6) studies identified by the present work for controlling castration pain, *Cortisol* will likely continue to be relied upon as pain research in cattle progresses.

#### 3.2.2. Meloxicam and Flunixin: Disbudding

**Figure 5:**
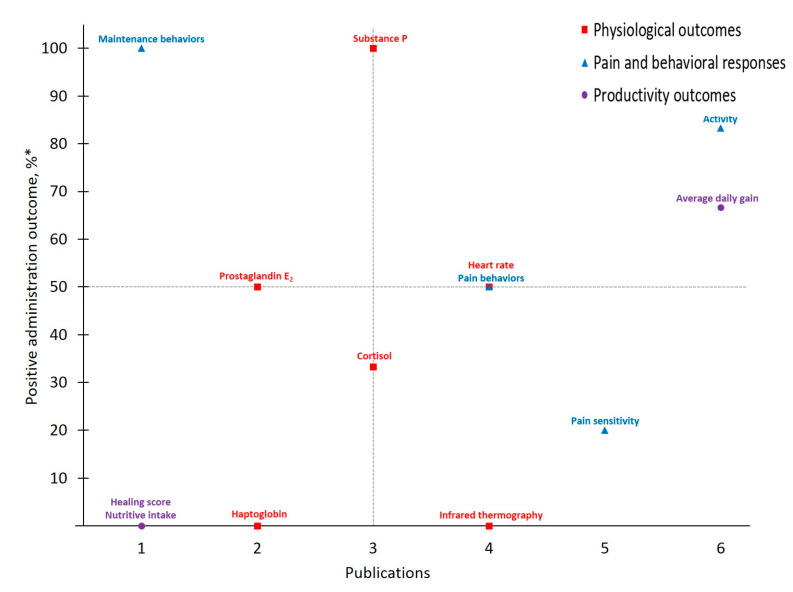
The number of publications that measured a given outcome and the percentage of publications that demonstrated meloxicam to have positive administration outcomes for cattle at disbudding. Positive administration outcome was determined based on efficacy statements made by authors of these publications as demonstrated by a *p*-value less than 0.05 between meloxicam and control treatment. ^*^ $\mathrm{Positive}\;\mathrm{administration}\;\mathrm{outcome}\;\%=\frac{\#\;\mathrm{of}\;\mathrm{publicationsthat}\;\mathrm{demonstrated}\;\mathrm{positive}\;\mathrm{administration}\;\mathrm{outcome}\;\mathrm{via}\;x\;\mathrm{outcome}}{\#\;\mathrm{of}\;\mathrm{publications}\;\mathrm{that}\;\mathrm{measured}\;x\;\mathrm{outcome}}\times 10$.

**Figure 6:**
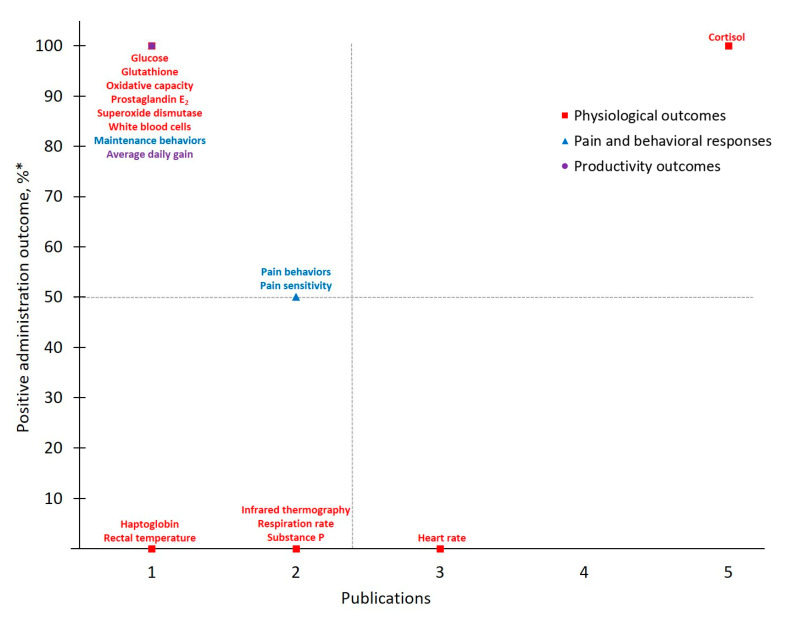
The number of publications that measured a given outcome and the percentage of publications that demonstrated flunixin to have positive administration outcomes for cattle at disbudding Positive administration outcome was determined based on efficacy statements made by authors of these publications as demonstrated by a *p*-value less than 0.05 between meloxicam and control treatment. ^*^ $\mathrm{Positive}\;\mathrm{administration}\;\mathrm{outcome}\;\%=\frac{\#\;\mathrm{of}\;\mathrm{publicationsthat}\;\mathrm{demonstrated}\;\mathrm{positive}\;\mathrm{administration}\;\mathrm{outcome}\;\mathrm{via}\;x\;\mathrm{outcome}}{\#\;\mathrm{of}\;\mathrm{publications}\;\mathrm{that}\;\mathrm{measured}\;x\;\mathrm{outcome}}\times 10$.

Data collected from publications that explored pain in cattle administered meloxicam (MEL-DISBUD; *n* = 15) or flunixin (FLU-DISBUD; *n* = 6) at disbudding are presented in Table 4 and Table 5, respectively. Eleven publications reported that meloxicam demonstrated positive administration outcomes in nine out of the 13 total outcomes measured when compared to control treatments for disbudding pain. All six publications identified by the present study reported that flunixin demonstrated positive administration outcomes in eleven out of 20 total outcomes measures when compared to control treatments fordisbudding pain. Publication totals and positive administration outcome rates for each measured outcome in disbudded cattle administered meloxicam or flunixin are presented in Figure 5 and Figure 6, respectively. Additionally, of the publications that reported calf age, 53% of studies investigating disbudding reported using animals ≤ two months of age, while 40 and 7% reported using calves between two and six months of age and > six months of age, respectively.

As with trends identified in the body of work for pain mitigation in cattle at castration, published investigations of disbudding pain in cattle considered meloxicam’s analgesic effects more frequently than flunixin. Of note, only 13 different measures were considered across MEL-DISBUD literature identified here and ***Physiological Outcomes*** (*n* = 18) and ***Pain and Behavioral Responses*** (*n* = 16) were measured with approximately equal frequency. However, of the four outcomes that demonstrated > 50% positive administration outcome rates, only one was categorically physiological (*Substance P* [42,47,57]) and overall behaviors were favored from a positive administration outcome standpoint. As with previous discussion, *Activity* stood out as yielding a higher positive administration outcome across MEL-DISBUD studies and was measured frequently. Interestingly, and in contrast to other drug-procedure combinations, average daily gain (*ADG*), a ***Productivity Outcome***, was measured as frequently as *Activity* and retained a positive administration outcome rate > 50% across MEL-DISBUD publications [42,47,58,59]. One such publication by Coetzee et al. [47] suggests that drug-associated differences in adrenal activity or bunk behaviors (e.g., greater time at the feed bunk; reported by Theurer et al. [66]) may contribute to this marked performance increase, though such appraisal remains beyond the scope of the present study.

Given that the number of publications for FLU-DISBUD was small, only limited information can be gleaned and any result interpretation or generalizations should proceed cautiously. For example, 13 of the 20 total measured outcomes were only measured by one publication and no published work exists, to date, against which to verify these reports. However, *Cortisol*, consistently among the most frequently measured outcomes identified by the present study, yielded a 100% positive administration outcome for this drug-procedure combination and was measured with the greatest frequency (*n* = 5) across the research available for FLU-DISBUD. Overall, more research for FLU-DISBUD is needed to draw strong conclusions and reliable, scientifically validated pain assessments should be used in any such future research.

#### 3.2.3. Aspirin

Only one study investigated aspirin in association with procedural pain in cattle and this information is not included in tables or figures. Briefly, aspirin was administered orally (50 mg/kg) to calves one minute prior to surgical castration and circulating concentrations of cortisol were measured [68]. Aspirin did not have any detectable effects on cortisol concentrations [68].

Aspirin represents a commonly used pharmaceutical option reduce fever, treat respiratory disease and provide pain relief for minor muscle and joint issues in non-lactating cattle [8]. Although sharing a lack of formal U.S. FDA approval with meloxicam, aspirin is sold over the counter and no veterinarian is required to obtain the drug [5]. Easier accessibility to aspirin, compared with other analgesics, helps keep input costs low for producers, which remains critical for the long-term viability of animal operations. In a 2017 report by Moggy et al. [69], a robust majority of surveyed cattle producers either ‘Strongly agreed’ or ‘Somewhat agreed’ that castration (70%) and disbudding (85%) procedures are painful. However, this and similar works also identified medication cost as a substantial barrier to producers providing pain management on-farm [7,69] and this challenge persists across livestock production industries [70]. Despite its practicality, the pharmacokinetic properties of aspirin also remain underreported and not well characterized in cattle.

### 3.3. Limitations and Opportunities

To date, the small size and notable variability of research available for meloxicam, flunixin and aspirin use in cattle undergoing castration or disbudding procedures continues to limit the development of recommendations for such use in cattle. Specifically, more research into aspirin’s PK profile is necessary given its wide use and on-farm practicality. In addition to limitations associated with the quantity of published research currently available, lack of reliable, scientifically validated methodologies for assessing pain in cattle inhibit progress toward identifying an effective drug and subsequently obtaining U.S. FDA approval. This challenge persists across livestock species and represents a growing area of research.

Given that no physiological biomarkers for pain have been reliably identified and agreed upon, many experts are choosing to explore behavioral pain assessments. For example, behavioral assessment methodologies were recently evaluated for quantifying behavioral deviations as indicators of pain in castrated piglets [71]. Moreover, this work uncovered only one method that could be validated amongst several techniques used in previous studies, highlighting the need for proactive validation of pain assessment methodologies [71]. Moving forward, refined and scientifically validated pain assessment methodologies for cattle are needed to facilitate reliable drug efficacy evaluation techniques required for continued progress in pain mitigation research and to obtain U.S. FDA approval of an effective drug for controlling procedural pain.

## 4. Conclusions

Regarding the body of work surrounding these three drug categories on pain mitigation for cattle, readers should interpret this data with caution. Firstly, overall publication number for castration and disbudding is low yielding a total of 25 and 21 peer-reviewed publications to date. Although a formal qualitative analysis of these publications was outside of the scope of this work, experimental design dissimilarities within these publications were pronounced, with variability noted in animal number, breed, and drug administration route and volume. These factors, in addition to author bias and interpretation of data, make it difficult to discern not only what outcomes are true indicators of pain control, but what drugs actually demonstrate true efficacy.

What these data do demonstrate to the reader is that more controlled research investigating pain mitigation in cattle is needed. In order for veterinarians to make meaningful recommendations for pain control on-farm, research must be conducted using a solid experimental design with an appropriate sample number if data are to be relied on. This, in addition to the lack of validated pain assessment methodologies in cattle, will be the most predominant limiting step that will prevent industry from making progressive steps to mitigate pan associated with common procedures.

## Figures and Tables

**Table 1:** Outcomes extracted and categorized.

Physiological Outcomes	Pain and Behavioral Responses	Productivity Outcomes
Acute phase response *Haptoglobin, serum amyloid A, substance P*	Activity *Exit velocity and chute activity, leg movement, lying, standing, walking*	Carcass characteristics *Backfat, hot carcass weight, kidney-pelvis-heart adjusted %, marbling, ribeye area, USDA yield grade*
Cardiovascular *Heart rate, heart rate variability*	Maintenance behaviors *Necessary activities, drinking, feeding, rumination*	Nutritive intake *Feed intake, milk consumption*
Complete blood counts *Hematocrit, hemoglobin, red and white blood cells, platelets*	Pain behaviors *Ear flicking, looking at wound, tail swishing/flicking*	Healing *Healing score, inflammation/swelling score, wound morphology*
Cortisol *Hair, plasma, salivary*	Pain sensitivity *Electronic reactivity measurements, maximum nociceptive threshold, wound sensitivity*	Weight *Average daily gain, bodyweight, gain:feed*
Cytokines *Interferons, interleukins, tumor necrosis factors*	Gait analysis *Stride length*	
Leukocytes *Basophils, eosinophils, lymphocytes, monocytes, neutrophils, neutrophils:lymphocytes*	Visual analog scale	
Prostaglandin E_2_	Vocalization	
Respiration rate		
Temperature *Ocular, rectal, scrotal, wound*		

**Table 2:** Pharmacokinetic profiles for meloxicam (*n* = 22) and flunixin (*n* = 44) based on data sets identified by the present study.

Route	Dose, mg/kg	T_1/2_, h		T_max,_ h		V_d_ or V_d_/F, L/kg		CL or CL/F, L/h/kg		*n*
		Min	Max	Min	Max	Min	Max	Min	Max	
Meloxicam										
IV	0.5	20.4	21.9	-	-	0.18	0.18	0.10	0.11	2
PO	0.5–1.0	11.9	40.0	11.3	24.0	0.16	0.38	0.09	0.22	16 *
SC	0.5	8.95	24.7	3.70	6.00	0.07	0.18	0.08	0.13	4
Flunixin										
IV	1.1-2.2	3.14	12.9	-	-	0.38	25.2	1.12	4.70	24
IM	1.1-2.2	1.87	15.5	0.25	3.10	-	-	-	-	9 *
PO	2.2	-	-	-	-	-	-	-	-	1 *
SC	2.0-2.2	4.50	7.46	1.10	3.47	-	-	-	-	4 *
TD	3.3	6.42	13.2	1.66	2.14	3.05	7.47	5.49	6.52	6 *

IV = intravenous; PO = *per os* (oral); SC = subcutaneous; IM = intramuscular; TD = transdermal; T_1/2_ = apparent terminal half-life; T_max_ = time at maximum concentration; V_d_ = volume of distribution; /F = per fraction absorbed; CL = clearance; *n* = number of datasets; * Excludes multiple dose studies.

**Table 3:** Results of publications that examined meloxicam and castration in cattle (*n* = 16).

Reference	Method	Administration			Outcomes Measured	Positive Administration Outcome ^1^
		Route	Dose	Timing		
Brown et al., 2015 [17]	Surgical *	PO	1.0mg/kg	U	Basophils and eosinophils, CBC, Hp, IFN, IL-6, monocytes, N:L, RT, TNF; Activity; ADG, carcass characteristics ^2^	Hp; Activity; ADG
Laurence et al., 2018 [18]	Surgical ^†^	SC	0.5 mg/kg	“pre-op” or “post-op”	C ^P^; Activity, baulk & chute scores; BW	C ^P^ (only when given “pre-op”)
Lehmann et al., 2017 [19]	Surgical	SC	0.5 mg/kg	30 m PRE	HR, mean arterial pressure; Somatic responses; BW	HR, mean arterial pressure
Marti et al., 2018 [20]	Surgical * or Band^*^	SC	0.5 mg/kg	U	CBC, C ^H^, Hp, N:L, RT, SAA, ScC, ScT; Maintenance & pain behaviors, stride length; BW, inflammation score	-
Marti et al., 2019 [21]	Surgical * (+/- Branding)	SC	0.5 mg/kg	IP	CBC, C ^H^, Hp, N:L, SAA, ScC, ScT; Lying/standing, pain behaviors, stride length, suckling; ADG, healing score	-
Melendez et al., 2018 [22]	Surgical * or Band^*^	SC	0.5 mg/kg	U	CBC, C ^S^, RT, SAA, ScT, SP; Leg movement, maintenance & pain behaviors, stride length, VAS, vocalization; ADG	CBC ^WBC^, SP; Lying time, tail flicking
Melendez et al., 2018 [23]	Surgical * (+/- Branding)	SC	0.5 mg/kg	IP	CBC, C ^S^, Hp, N:L, RT, SAA, SP, ScT; Activity, electronic reactivity measurements, leg movement, pain behaviors, stride length, VAS, vocalization; ADG	CBC ^WBC^, N:L, ScT; Leg movement, lying time, tail flicking, walking time
Melendez et al., 2018 [24]	Surgical ^†^	SC	0.5 mg/kg	30 m PRE	C ^H,S^, Hp, RT, SAA, ScC, ScT, SP, WBC; Activity, leg movement, maintenance & pain behaviors, stride length, VAS, vocalizations; ADG	C ^S^, Hp, ScT, WBC
Musk et al., 2017 [25]	Surgical	SC	0.5 mg/kg	30 m PRE	Fe, fibrinogen, Hp, SAA; Activity; BW	-
Musk et al., 2017 [26]	Surgica l ^†^	SC	0.5 mg/kg	30m PRE^*^ or “immediately after” ^†^	Mechanical nociceptive threshold	-
Olson et al., 2016 [27]	Surgical *	PO	1.0 mg/kg	2 h PRE	C ^P^, HR, substance P; Activity, pain behaviors, tail movement, VAS; Inflammation score	C ^P^, HR, substance P; Activity, VAS; Inflammation score
	or Band^*^					
Repenning et al., 2013 [28]	Band *	PO	1.0 mg/kg, 0.2 mg/kg, 0.5 mg/kg	24 h PRE, 0 h, 24 h POST	Rectal temperature; Chute behaviors, maintenance behaviors; ADG, dry matter intake, gain:feed	Rectal temperature; Bunk and standing behaviors
Roberts et al., 2015 [29]	Surgical *	PO	1.0 mg/kg	“concurrent with procedure”	CBC, C ^P^, cytokines, glucose, Hp, leukocytes, non-esterified fatty acids, rectal temperature	CBC ^RBC,WBC^, C ^P^, eosinophils, Hp, monocytes, neutrophils
Roberts et al., 2018 [30]	Surgical * or Band *	PO	15 mg/calf	“concurrent with procedure”	Hp; Activity; ADG, carcass characteristics	Activity; ADG, back fat, ribeye area
Sutherland et al., 2019 [31]	Band *	PO	50mg/calf	IP	Respiration rate; Activity, environmental interactions, pain behaviors, rumination	-
Vindevoghel et al., 2019 [32]	Surgical	SC	0.5 mg/kg	“immediately after”	Maintenance, pain and social behaviors, qualitative behavioral assessment score	Qualitative behavioral assessment score

* Procedure performed without anesthesia; **^†^** Variable anesthesia use between treatments; PO = per os (oral); SC = subcutaneous; U = unspecified; IP = “immediately prior”; pre- or post-op = before or after the operation/procedure; PRE = before the procedure; POST = after the procedure ^1^ Positive administration outcome was determined based on efficacy statements made by authors of these publications as demonstrated by a *P*-value less than 0.05 between meloxicam and control treatments. Outcomes Measured: ADG = average daily gain; BW = body weight; C = cortisol (^H^Hair; ^P^Plasma; ^S^Salivary); CBC = complete blood count (^WBC^White blood cell count); Hp = haptoglobin; HR = heart rate; IFN = interferon gamma; IL-6 = interleukin-6; N:L = neutrophil:lymphocyte; RT = rectal temperature; SAA = serum amyloid A; SP = substance P; ScC = scrotal circumference; ScT = scrotal temperature; TNF = tumor necrosis factor; VAS = visual analog scale.

**Table 4:** Results of publications that examined flunixin in cattle at castration and disbudding procedures.

Reference	Method	Administration			Outcomes Measured	Positive Administration Outcome ^1^
		Route	Dose	Timing		
Castration (*n* = 9)						
Currah et al., 2009 [33]	Surgical	IV	2.2 mg/kg	U	Activity, nursing, stride length, visual pain assessment, vocalization	Stride length
Kleinhenz et al., 2018 [34]	Surgical	TD	3.3 mg/kg	“during”	C ^P^, HR, IRT ^O^, substance P; Gait analysis, stride length	C ^P^
Mintline et al., 2014 [35]	Surgical	IV	1.1 mg/kg	IP	IRT ^W^, scrotal circumference & temperature, substance P; Lying time; ADG, healing score	-
Park et al., 2018 [36]	Surgical	IV	2.0 mg/kg	IP	C ^P^, glutamic oxaloacetic and pyruvate transaminases, Hp, RT, SP; ADG, feed intake	Haptoglobin, RT
Paull et al., 2015 [37]	Band	SC	200 mg	IA	Complete blood count, C ^P^, haptoglobin, leukocyte counts; Pain behaviors, postures; ADG	-
Repenning et al., 2013 [28]	Surgical or Band	IV	1.2 mg/kg	“upon restraint” + days 1, 2, 3	HR, RR, RT; Activity, feeding, procedure response score; ADG, DMI, G:F	RR; Activity (chute behaviors), feeding, procedure response score
Stilwell et al., 2008 [38]	Surgical	SC	2.2 mg/kg	5 m PRE	C ^P^; Bunk behaviors, pain behaviors, vocalizations	C ^P^
Sutherland et al., 2013 [39]	Surgical	IM	2.0 mg/kg	IP	C ^P^, leukocytes, WBC; Activity, maintenance & pain behaviors	C ^P^; Drinking and eating
Webster et al., 2013 [40]	Surgical ^†^	IV	1.1 mg/kg	20 m PRE	C ^P^; Activity, maintenance & pain behaviors, postures	C ^P^; Postures
Disbudding (*n* = 6)						
Fraccaro et al., 2013 [41]	Surgical	IV	2.2 mg/kg	IP	Prostaglandin E_2_	Prostaglandin E_2_
Glynn et al., 2013 [42]	Scoop	IV	2.2 mg/kg	1 m PRE	C ^P^, haptoglobin, IRT ^O^, substance P; MNT; ADG	C ^P^; ADG
Huber et al., 2013 [43]	Hot iron	IV	2.2 mg/kg	IP or IP + 3 h POST	C ^P^, HR, RR; Pain behaviors	C ^P^ (only when given 2×)
Kleinhenz et al., 2017 [44]	Hot iron *	TD	3.3 mg/kg	Concurrent	C ^P^, HR, IRT ^O^, substance P; MNT	C ^P^; MNT
Sutherland et al., 2013 [39]	Surgical	IM	2.0 mg/kg	IP	C ^P^, leukocytes, WBC; Activity, maintenance & pain behaviors	C ^P^, WBC; Eating, headshake
Yakan et al., 2018 [45]	Paste	IV	2.2 mg/kg	15 m PRE	C ^P^, glucose, glutathione, HR, oxidative capacities, RR, RT, superoxide dismutase	C ^P^, glucose, glutathione, superoxide dismutase, antioxidant capacity

* Procedures performed without anesthesia; **^†^**Variable anesthesia use between treatments; IV = intravenous; TD = transdermal; U = unspecified; IP = “immediately prior”; IA = “immediately after”. Outcomes Measured: ADG = average daily gain; C ^P^ = cortisol; DMI = dry matter intake; G:F = gain:feed; HR = heart rate; IRT = infrared thermography (^O^ Ocular; ^W^ Wound); MNT = maximum nociceptive threshold; RT = rectal temperature; RR = respiration rate; WBC = white blood cells. ^1^ Positive administration outcome was determined based on efficacy statements made by authors of these publications as demonstrated by a *p*-value less than 0.05 between flunixin and control treatment.

**Table 5:** Results of studies that have examined meloxicam use to mitigate pain in cattle at disbudding (*n* = 15).

Reference	Method	Administration			Outcomes Measured	Positive Administration Outcome *
		Route	Dose	Timing		
Allen et al., 2013 [57]	Hot iron	PO	1.0 mg/kg	12 h PRE or “immediately following”	C *, haptoglobin, IRT *, prostaglandin E_2_, SP; MNT; ADG	C *, prostaglandin E_2_ (only when given “immediately following”), SP
Bates et al., 2015 [58]	Hot iron	SC	20 mg/calf	IP	ADG, milk consumption	ADG
Bates et al., 2016 [59]	Hot iron	SC	20 mg/calf	“at the time of disbudding”	ADG	ADG
Byrd et al., 2019 [60]	Hot iron	PO	15 mg/calf	15 m PRE	HRV	-
Clapp et al., 2015 [61]	Hot iron	SC	0.5 mg/kg	U	HRV; Activity (stress behaviors), approach test	-
Coetzee et al., 2012 [47]	Hot iron *	IV	0.5 mg/kg	U	C *, HR, SP; Activity; ADG	ADG, HR, SP; Activity
Cuttance et al., 2019 [62]	Hot iron *	SC	0.5 mg/kg	10 m PRE	Pain behaviors, pain sensitivity; ADG	Ear flicking and head scratching(only when given with anesthetic)
Ede et al., 2019 [63]	Hot iron	U	U	U	Pen conditioning activity	Lying bouts, time in pen
Fraccaro et al., 2013 [41]	Surgical	PO	1.0 mg/kg	IP	Prostaglandin E_2_	-
Glynn et al., 2013 [42]	Scoop	PO	2.2 mg/kg	1 m PRE	C *, haptoglobin, IRT *, SP; MNT; ADG	ADG, SP
Heinrich et al., 2010 [64]	Hot iron	IM	0.5 mg/kg	10 m PRE	Activity, MNT, pain behaviors, pain sensitivity	Activity, ear flicking and head shaking, pain sensitivity
Mintline et al., 2013 [65]	Hot iron	IV	0.5 mg/kg	55 m PRE	Play behaviors, wound sensitivity	Bucking and running behaviors
Stewart et al., 2009 [46]	Hot iron	IV	0.5 mg/kg	30 m PRE	HR, HRV, IRT *	HR, HRV
Theurer et al., 2012 [66]	Hot iron *	PO	0.5 mg/kg	“immediately after”	Activity, maintenance behaviors	Drinking and feeding behaviors, lying time
Van der Saag et al., 2018 [67]	Scoop *	PO	0.5 mg/kg	25 m PRE	IRT *; Activity, pain behaviors; Wound morphology	-

* Procedures performed without anesthesia; **^†^** Variable anesthesia use between treatments; PO = per os (oral); SC = subcutaneous; IV = intravenous; IM = intramuscular; U = unspecified; IP = “immediately prior”. ^1^Positive administration outcome was determined based on efficacy statements made by authors of these publications as demonstrated by a *p*-value less than 0.05 between meloxicam and control treatment. Outcomes Measured: ADG = average daily gain; C^P^ = cortisol (plasma); HR = heart rate; HRV = heart rate variability; IRT = infrared thermography (^O^Ocular; ^W^Wound); MNT = maximum nociceptive threshold; SP = substance P.

## Data Availability

Publicly available datasets were analyzed in this study. The data can be found via their citation information presented in full in the ‘References’ section of the present publication.

## Appendix A

**Table A1:** Specific inclusion and exclusion criteria used in Covidence online platform to flag key words to assist with manual screening of publication titles and abstracts.

Inclusion Criteria	Exclusion Criteria
✓ Analgesia; analgesic(s) ✓ Aspirin ✓ Boar(s) * ✓ Calf; calves ✓ Castration ✓ Cattle ✓ Cow(s) ✓ Dehorning ✓ Disbudding ✓ Flunixin (meglumine) ✓ Gilt(s) * ✓ Heifer(s) ✓ Meloxicam ✓ Non(-)steroidal anti(-)inflammatory drug(s); NSAID(s) ✓ Pain(ful) ✓ Pig(s); piglet(s) * ✓ Post(-)surgical ✓ Procedur(e)(s)/(al) ✓ Process(ing) ✓ Sensitivity ✓ Swine * ✓ Tail docking *	⊗ Cell(s) ⊗ DNA ⊗ Goat(s) ⊗ Guinea * ⊗ Horse(s) ⊗ Human(s) ⊗ In situ ⊗ Lamb(s) ⊗ Mice ⊗ Mouse ⊗ Patient(s) ⊗ Rat(s) ⊗ Sheep

* Terms included in this initial “Title and Abstract” screening process specifically relevant to the swine-based portion of this work not presented in this manuscript.

## References

[1] Adcock S.J.J., Tucker C.B., Tucker C.B. (2018). Painful procedures: When and what should we be measuring in cattle. Advances in Cattle Welfare.

[2] Coetzee J.F., Nutsch A.L., Barbur L.A., Bradburn R.M. (2010). A survey of castration methods and associated livestock management practices performed by bovine veterinarians in the United States. BMC Vet. Res..

[3] Fajt V.R., Wagner S.A., Norby B. (2011). Analgesic drug administration and attitudes about analgesia in cattle among bovine practitioners in the United States. J. Am. Vet. Med. Assoc..

[4] Johnstone E.C.S., Coetzee J.F., Pinedo P.J., Edwards-Callaway L. (2021). Survey investigating current attitudes towards use of pain mitigation practices in beef and dairy cattle in the US by veterinarians and producers. J. Am. Vet. Med. Assoc..

[5] Schmitz A. Progressive Dairy. https://www.progressivedairy.com/topics/herd-health/how-to-solve-the-lack-of-medications-labeled-for-cow-pain-relief.

[6] Engle T.E., Klingborg D.J., Rollin B.E. (2019). The Welfare of Cattle.

[7] Robles I., Arruda A., Nixon E., Johnstone E., Wagner B.K., Edwards-Callaway L., Baynes R.E., Coetzee J.F., Pairis-Garcia M.D. (2021). Producer and veterinarian perspectives towards pain management practices in the US cattle industry. Animals.

[8] Coetzee H. (2017). Pharmocological approaches to pain management in cattle. J. Anim. Sci..

[9] Smith G.W., Davis J.L., Tell L.A., Webb A.I., Riviere J.E. (2008). Extralabel use of nonsteroidal anti-inflammatory drugs in cattle. J. Am. Vet. Med. Assoc..

[10] AMDUCA Animal Medicinal Drug Use Clarification Act of 1994 (AMDUCA). https://www.fda.gov/animal-veterinary/acts-rules-regulations/animal-medicinal-drug-use-clarification-act-1994-amduca.

[11] Riviere J.E., Martin-Jiminez T., Sundlof S.F., Craigmill A.L. (1997). Interspecies allometric analysis of the comparative pharmacokinetics of 44 drugs across veterinary and laboratory animal species. J. Vet. Pharmacol. Ther..

[12] Riviere J.E., Sundlof S.F., Craigmill A.L. (1991). Handbook of Comparative Pharmacokinetics and Residues of Veterinary Antimicrobials. Boca Raton.

[13] Teixera R., Valberg S. The Risk of Administration of Intramuscular Drugs such as Banamine® to Horses. https://www.vmc.umn.edu/sites/vmc.umn.edu/files/the-risk-of-administration-of-intramuscular-drugs-such-as-banamine-to-horses.pdf.

[14] Rang H.P., Ritter J.M., Flower R.J., Henderson G. (2016). Rang & Dale’s Pharmacology.

[15] Martinez M.N., Court M.H., Fing-Gremmels J., Mealey K.L. (2018). Population variability in animal health: Influence on dose–exposure–response relationships: Part I: Drug metabolism and transporter systems. J. Vet. Pharmacol. Ther..

[16] Packiasabapathy S., Sadhasivam S. (2018). Review: Gender, genetics, and analgesia: Understanding the differences in response to pain relief. J. Pain Res..

[17] Brown A.C., Powell J.G., Kegley E.B., Gadberry M.S., Reynolds J.L., Hughes H.D., Carroll J.A., Sanchez N.C.B., Thaxton Y.V., Backes E.A. (2015). Effect of castration timing and oral meloxicam administration on growth performance, inflammation, behavior, and carcass quality of beef calves. J. Anim. Sci..

[18] Laurence M., Barnes A., Collins T., Hyndman T., Musk G.C. (2018). Assessing and mitigating post-operative castration pain in *Bos indicus* cattle. Anim. Prod. Sci..

[19] Lehmann H.S., Musk G.C., Laurence M., Hyndman T.H., Tuke J., Collins T., Gleerup K.B., Johnson C.B. (2017). Mitigation of electroencephalographic and cardiovascular responses to castration in Bos indicus bulls following the administration of either lidocaine or meloxicam. Vet. Anaesth. Analg..

[20] Marti S., Meléndez D.M., Pajor E.A., Moya D., Gellatly D., Janzen E.D., Schwartzkopf-Genswein K.S. (2018). Effect of a single dose of subcutaneous meloxicam prior to band or knife castration in 1-wk-old beef calves: II. Inflammatory response and healing. J. Anim. Sci..

[21] Marti S., Meléndez D.M., Pajor E.A., Moya D., Gellatly D., Janzen E.D., Schwartzkopf-Genswein K.S. (2019). Effect of a single dose of subcutaneous meloxicam before knife castration alone or combined with hot-iron branding on scrotal healing, inflammatory response, and behaviour in 2-mo-old beef calves over 42 d post procedure. Can. J. Anim. Sci..

[22] Meléndez D.M., Marti S., Pajor E.A., Moya D., Gellatly D., Janzen E.D., Schwartzkopf-Genswein K.S. (2018). Effect of a single dose of meloxicam prior to band or knife castration in 1-wk-old beef calves: I. Acute pain. J. Anim. Sci..

[23] Meléndez D.M., Marti S., Pajor E.A., Moya D., Gellatly D., Janzen E.D., Schwartzkopf-Genswein K.S. (2018). Effect of subcutaneous meloxicam on indicators of acute pain and distress after castration and branding in 2-mo-old beef calves. J. Anim. Sci..

[24] Meléndez D.M., Marti S., Pajor E.A., Sidhu P.K., Gellatly D., Moya D., Janzen E.D., Coetzee J.F., Schwartzkopf-Genswein K.S. (2018). Effect of meloxicam and lidocaine administered alone or in combination on indicators of pain and distress during and after knife castration in weaned beef calves. PLoS ONE.

[25] Musk G.C., Jacobsen S., Hyndman T.H., Lehmann H.S., Tuke S.J., Collins T., Gleerup K.B., Johnson C.B., Laurence M. (2017). Objective measures for the assessment of post-operative pain in *Bos indicus* bull calves following castration. Animals.

[26] Musk G.C., Laurence M., Collins T., Tuke J., Hyndman T.H. (2017). Mechanical nociceptive threshold testing in *Bos indicus* bull calves. Anim. Prod. Sci..

[27] Olson M.E., Ralston B., Burwash L., Matheson-Bird H., Allan N.D. (2016). Efficacy of oral meloxicam suspension for prevention of pain and inflammation following band and surgical castration in calves. BMC Vet. Res..

[28] Repenning P.E., Ahola J.K., Callan R.J., Fox J.T., French J.T., Giles R.L., Peel R.K., Whittier J.C., Engle T.E. (2013). Effects of pain mitigation and method of castration on behavior and feedlot performance in cull beef bulls. J. Anim. Sci..

[29] Roberts S.L., Hughes H.D., Sanchez N.C.B., Carroll J.A., Powell J.G., Hubbell D.S., Richeson J.T. (2015). Effect of surgical castration with or without oral meloxicam on the acute inflammatory response in yearling beef bulls. J. Anim. Sci..

[30] Roberts S.L., Powell J.G., Hughes H.D., Richeson J.T. (2018). Effect of castration method and analgesia on inflammation, behavior, growth performance, and carcass traits in feedlot cattle. J. Anim. Sci..

[31] Sutherland M.A., Bright A.L., Schuetz K.E. (2019). Effect of a buccal meloxicam formulation on the behavioural response to ring castration of calves. Anim. Prod. Sci..

[32] Vindevoghel T.V., Fleming P.A., Hyndman T.H., Musk G.C., Laurence M., Collins T. (2019). Qualitative Behavioural Assessment of *Bos indicus* cattle after surgical castration. Appl. Anim. Behav. Sci..

[33] Currah J.M., Hendrick S.H., Stookey J.M. (2009). The behavioral assessment and alleviation of pain associated with castration in beef calves treated with flunixin meglumine and caudal lidocaine epidural anesthesia with epinephrine. Can. Vet. J..

[34] Kleinhenz M.D., Van Engen N.K., Smith J.S., Gorden P.J., Ji J., Wang C., Perkins S.C.B., Coetzee J.F. (2018). The impact of transdermal flunixin meglumine on biomarkers of pain in calves when administered at the time of surgical castration without local anesthesia. Livest. Sci..

[35] Mintline E.M., Varga A., Banuelos J., Walker K.A., Hoar B., Drake D., Weary D.M., Coetzee J.F., Stock M.L., Tucker C.B. (2014). Healing of surgical castration wounds: A description and an evaluation of flunixin. J. Anim. Sci..

[36] Park S.J., Piao M., Kim H., Kang H.J., Seo J., Lee S., Baik M. (2018). Effects of castration and a lidocaine-plus-flunixin treatment on growth and indicators of pain, inflammation, and liver function in Korean cattle bull calves. Livest. Sci..

[37] Paull D.R., Small A.H., Lee C., Labeur L., Colditz I.G. (2015). Effect of local infusion of NSAID analgesics administered alone or in combination on the pain associated with band castration in calves. Aust. Vet. J..

[38] Stilwell G., Lima M.S., Broom D.M. (2008). Effects of nonsteroidal anti-inflammatory drugs on long-term pain in calves castrated by use of an external clamping technique following epidural anesthesia. Am. J. Vet. Res..

[39] Sutherland M.A., Ballou M.A., Davis B.L., Brooks T.A. (2013). Effect of castration and dehorning singularly or combined on the behavior and physiology of Holstein calves. J. Anim. Sci..

[40] Webster H.B., Morin D., Jarrell V., Shipley C., Brown L., Green A., Wallace R., Constable P.D. (2013). Effects of local anesthesia and flunixin meglumine on the acute cortisol response, behavior, and performance of young dairy calves undergoing surgical castration. J. Dairy Sci..

[41] Fraccaro E., Coetzee J.F., Odore R., Edwards-Callaway L.N., KuKanich B., Badino P., Bertolotti L., Glynn H., Dockweiler J., Allen K. (2013). A study to compare circulating flunixin, meloxicam and gabapentin concentrations with prostaglandin E_2_ levels in calves undergoing dehorning. Res. Vet. Sci..

[42] Glynn H.D., Coetzee J.F., Edwards-Callaway L.N., Dockweiler J.C., Allen K.A., Lubbers B., Jones M., Fraccaro E., Bergamasco L.L., KuKanich B. (2013). The pharmacokinetics and effects of meloxicam, gabapentin, and flunixin in postweaning dairy calves following dehorning with local anesthesia. J. Vet. Pharmacol. Ther..

[43] Huber J., Arnholdt T., Moestl E., Gelfert C.C., Drillich M. (2013). Pain management with flunixin meglumine at dehorning of calves. J. Dairy Sci..

[44] Kleinhenz M.D., Van Engen N.K., Gorden P.J., Ji J., Walsh P., Coetzee J.F. (2017). Effects of transdermal flunixin meglumine on pain biomarkers at dehorning in calves. J. Anim. Sci..

[45] Yakan S., Duzguner V., Aksoy O. (2018). Effects of flunixin meglumine on oxidant and antioxidant system after disbudding with caustic paste in calves. Acta. Sci. Vet..

[46] Stewart M., Stookey J.M., Stafford K.J., Tucker C.B., Rogers A.R., Dowling S.K., Verkerk G.A., Schaefer A.L., Webster J.R. (2009). Effects of local anesthetic and a nonsteroidal antiinflammatory drug on pain responses of dairy calves to hot-iron dehorning. J. Dairy Sci..

[47] Coetzee J.F., Mosher R.A., KuKanich B., Gehring R., Robert B., Reinbold J.B., White B.J. (2012). Pharmacokinetics and effect of intravenous meloxicam in weaned Holstein calves following scoop dehorning without local anesthesia. BMC Vet. Res..

[48] Tucker C.B., Mintline E.M., Banuelos J., Walker K.A., Hoar B., Varga A., Drake D., Weary D.M. (2014). Pain sensitivity and healing of hot-iron cattle brands. J. Anim. Sci..

[49] Kumar A., Hancke G.P. (2015). A Zigbee-Based Animal Health Monitoring System. IEEE Sens. J..

[50] Molony V., Kent J.E. (1997). Assessment of Acute Pain in Farm Animals Using Behavioral and Physiological Measurements. J. Anim. Sci..

[51] Fraser D., Weary D.M., Pajor E.A., Milligan B.N. (1997). A Scientific Conception of Animal Welfare that Reflects Ethical Concerns. Anim. Welf..

[52] Appleby M.C., Olsson I.A.S., Galindo F. (2018). Animal Welfare.

[53] Tsigos C., Chrousos G.P. (2002). Hypothalamic–pituitary–adrenal axis, neuroendocrine factors and stress. J. Psychosom. Res..

[54] Anderson D.E., Muir W.W. (2005). Pain Management in Ruminants. Vet. Clin. Food Anim..

[55] Desborough J.P. (2000). The stress response to trauma and surgery. Brit. J. Anaesth..

[56] Stock M.L., Coetzee J.F. (2015). Clinical Pharmacology of Analgesic Drugs in Cattle. Vet. Clin. Food Anim..

[57] Allen K.A., Coetzee J.F., Edwards-Callaway L.N., Glynn H., Dockweiler J., KuKanich B., Lin H., Wang C., Fraccaro E., Jones M. (2013). The effect of timing of oral meloxicam administration on physiological responses in calves after cautery dehorning with local anesthesia. J. Dairy Sci..

[58] Bates A.J., Eder P., Laven R.A. (2015). Effect of analgesia and anti-inflammatory treatment on weight gain and milk intake of dairy calves after disbudding. N. Z. Vet. J..

[59] Bates A.J., Laven R.A., Chapple F., Weeks D.S. (2016). The effect of different combinations of local anaesthesia, sedative and non-steroidal anti-inflammatory drugs on daily growth rates of dairy calves after disbudding. N. Z. Vet. J..

[60] Byrd C.J., Craig B.A., Eicher S.D., Radcliffe J.S., Lay D.C. (2019). Short communication: Assessment of disbudding pain in dairy calves using nonlinear measures of heart rate variability. J. Dairy Sci..

[61] Clapp J.B., Croarkin S., Dolphin C., Lyons S.K. (2015). Heart rate variability: A biomarker of dairy calf welfare. Anim. Prod. Sci..

[62] Cuttance E.L., Mason W.A., Yang D.A., Laven R.A., McDermott J., Inglis K. (2019). Effects of a topically applied anaesthetic on the behaviour, pain sensitivity and weight gain of dairy calves following thermocautery disbudding with a local anaesthetic. N. Z. Vet. J..

[63] Ede T., von Keyserlingk M.A.G., Weary D.M. (2019). Assessing the affective component of pain, and the efficacy of pain control, using conditioned place aversion in calves. Biol. Lett..

[64] Heinrich A., Duffield T.F., Lissemore K.D., Millman S.T. (2010). The effect of meloxicam on behavior and pain sensitivity of dairy calves following cautery dehorning with a local anesthetic. J. Dairy Sci..

[65] Mintline E.M., Stewart M., Rogers A.R., Cox N.R., Verkerk G.A., Stookey J.M., Webster J.R., Tucker C.B. (2013). Play behavior as an indicator of animal welfare: Disbudding in dairy calves. Appl. Anim. Behav. Sci..

[66] Theurer M.E., White B.J., Coetzee J.F., Edwards L.N., Mosher R.A., Cull C.A. (2012). Assessment of behavioral changes associated with oral meloxicam administration at time of dehorning in calves using a remote triangulation device and accelerometers. BMC Vet. Res..

[67] Van der Saag D., Lomax S., Windsor P.A., Taylor C., White P.J. (2018). Evaluating treatments with topical anaesthetic and buccal meloxicam for pain and inflammation caused by amputation dehorning of calves. PLoS ONE.

[68] Coetzee J.F., Gehring R., Bettenhausen A.C., Lubbers B.V., Toerber S.E., Thomson D.U., Kukanich B., Apley M.D. (2007). Attenuation of acute plasma cortisol response in calves following intravenous sodium salicylate administration prior to castration. J. Vet. Pharmacol. Ther..

[69] Moggy M.A., Pajor E.A., Thurston W.E., Parker S., Greter A.M., Schwartzkopf-Genswein K.S., Campbell J.R., Windeyer M.C. (2017). Management practices associated with pain in cattle on western Canadian cow-calf operations: A mixed methods study. J. Anim. Sci..

[70] Wagner B., Royal K., Park R., Pairis-Garcia M. (2020). Identifying Barriers to Implementing Pain Management for Piglet Castration: A Focus Group of Swine Veterinarians. Animals.

[71] Park R.M., Cramer M.C., Wagner B.K., Turner P., Moraes L.E., Viscardi A.V., Coetzee J.F., Pairis-Garcia M.D. (2020). A comparison of behavioural methodologies utilised to quantify deviations in piglet behaviour associated with castration. Anim. Welf..

